# Cancer associated macrophage-like cells in metastatic renal cell carcinoma predicts for poor prognosis and tracks treatment response in real time

**DOI:** 10.1038/s41598-023-37671-3

**Published:** 2023-06-29

**Authors:** Amama Ali, Daniel L. Adams, Dimpal M. Kasabwala, Cha-Mei Tang, Thai H. Ho

**Affiliations:** 1 Creatv Bio, Division of Creatv MicroTech, Inc., 9 Deer Park Dr, Monmouth Junction, NJ 08852 USA; 2 Creatv Bio, Division of Creatv MicroTech, Inc., 9900 Belward Campus Dr., Rockville, MD 20850 USA; 3 Division of Hematology/Oncology, Mayo Clinic Arizona, 5777 East Mayo Boulevard, Phoenix, AZ 85054 USA

**Keywords:** Cancer, Renal cell carcinoma

## Abstract

Renal Cell Carcinoma (RCC) is a fatal urological cancer, with one third of patients diagnosed with metastasis, resulting in a 5-year survival of only 12%. Recent advancements in therapies have increased survival in mRCC, but lack efficacy in subtypes, due to treatment resistance and toxic side effects. Currently, white blood cells, hemoglobin, and platelets are limitedly used as blood based biomarkers to help determine RCC prognosis. Cancer associated macrophage-like cells (CAMLs) are a potential mRCC biomarker which have been identified in peripheral blood of patients with malignant tumors and have been shown to predict poor clinical patient outcomes based on their number and size. In this study, blood samples from 40 RCC patients were obtained to evaluate the clinical utility of CAMLs. CAML changes were monitored during treatment regimens to evaluate their ability to predict treatment efficacy. It was observed that patients with smaller CAMLs had better progression free survival (HR = 2.84, 95% CI 1.22–6.60, p = 0.0273) and overall survival (HR = 3.95, 95% CI 1.45–10.78, p = 0.0154) versus patients with larger CAMLs. These findings suggest that CAMLs can be used as a diagnostic, prognostic, and predictive biomarker for patients with RCC which may help improve management of advanced RCC.

## Introduction

Renal cell carcinoma (RCC), the 8th most common cancer, forms from renal tubular epithelial cells and makes up over 80% of all kidney cancers, with clear cell RCC being the largest subtype^1,2^. RCC affects ~ 79,000 individuals in the United States and results in ~ 13,900 deaths annually with incidence in men being 50% higher than in women and more common in people over the age of 60 years old^3,4^. The most common genetic cause for RCC is a disease of the Von-Hippel Lindau gene, while established non-genetic factors include smoking, obesity, hypertension, and chronic kidney disease^4^. In the past two decades, approximately 50% of RCC diagnoses were made due to incidental detection through newer imaging technologies resulting in ~ 33% RCC diagnoses being made at the metastatic stage^4^.

The International mRCC Database Consortium (IMDC) risk calculator is a well-established tool which can help predict survival outcomes in mRCC patients^3,5^. The IMDC uses multiple factors (hemoglobin level, time from diagnosis to start of treatment, neutrophil count, etc.) to determine whether a patient is in a good, intermediate, or a poor-risk grouping^3,5^. The patient’s categorical estimate can help doctors determine the type of first line therapy to use on patients^5^. To treat RCC, surgery is still the first choice for treating non-metastasized disease, while systemic targeted therapies (i.e. Pazopanib, Axitinib, etc.), and more currently PD-1 inhibiting immunotherapies (i.e. Nivolumab, Ipilimumab, etc.), are used as neoadjuvant therapy prior to surgery in the metastatic setting^6,7^. The recent addition of PD-1 immunotherapies have helped many patients with mRCC, i.e. the addition of Nivolumab and Ipilimumab in systemic treatment have increased the 18 month overall survival (OS) to 75% compared 60% with Sunitinib alone, in patients with poor prognostic risk^8^. However, a high number of patients have shown primary or adaptive resistance, as well as high grade toxicity, to these treatments which can be caused by patient-intrinsic factors, tumor cell-intrinsic factors, or tumoral microenvironment related factors^9–11^. Predictive biomarkers, such as tumor and stromal cell PD-L1 expression, are used to predict for a patients’ response or lack of response to therapy^12–14^. These biomarkers have been shown to help efficiently determine treatment pathways for individuals based on initial treatment response without the need to use imaging techniques therefore shortening the time needed to change treatments if necessary^12^. Stopping an ineffective treatment on a patient quickly can help decrease the likelihood of toxic side effects as well as reducing progression and prolonging a patient’s overall survival. While PD-L1 tumor/stromal expression is a currently used predictive biomarker, it is not highly accurate at predicting response to anti PD-1 therapies, as PD-L1 is a dynamic immune modulating biomarker that can change over time and upregulate in certain treatment types^13^. White blood cell (WBC) analysis such as high neutrophil count, low hemoglobin, and high platelet count all play a role in determining patient risk categorization using the IMDC risk calculator, but their use is limited to patients with untreated mRCC or patients with mRCC treated with first line targeted therapies, leaving a need to find more accurate predictive and prognostic biomarkers^12^.

Cancer associated macrophage like-cells (CAMLs) were recently identified in the peripheral blood of individuals with active malignancies, demonstrating the potential to be a valuable diagnostic and prognostic biomarker in a number of cancer subtypes^15,16^. CAMLs are myeloid cells that contain phagocytosed elements of the primary tumor and are found in all stages of cancer and multiple different cancer types^15–18^. CAMLs are not present in the blood of patients with benign conditions but have been linked to poor prognosis in patients with pancreatic, prostate and breast cancer^15–22^. Previous studies have shown that CAML changes can correspond to tumor response to treatment induction, however, it is unknown if CAMLs can be used to help determine treatment efficacy in RCC patients. Studies have shown that prolonged survival and better health have a correlation with smaller CAML size and that healthy patients do not have any CAMLs in their blood^15–22^. By tracking changes in CAML size before the start, during, and after treatment induction, it has been hypothesized that decreases in CAMLs may correlate to the effectiveness of treatment, whereas an increase would indicate treatment inefficacy. Further, blood based biopsies may allow for the monitoring of patients before the start and after induction of a new treatment, thereby quickly determining response to new therapy induction, which could help doctors personalize follow up treatment pathways for individual patients.

## Materials and methods

### Study design and patient population

A single blind prospective pilot study was conducted to evaluate the diagnostic and prognostic value of CAMLs in patients (N = 40) with RCC over a 2-year period. This study was run through an agreement with Mayo Clinic Cancer Center, with written informed consent with local Institutional Review Board (IRB) approval from Mayo Clinic Cancer Center. All research was performed in accordance with relevant guidelines and regulations and in accordance with the Declaration of Helsinki. Whole peripheral blood samples (7.5 mL) were collected from 40 anonymized patients with pathologically confirmed renal cell carcinoma. A 7.5 mL volume of blood has been set as the standard by the FDA, and is used in the CellSearch system, the only FDA approved CTC detection technique^28–31^. The number of patients used in this study was determined by using posterior power analysis. We sought to achieve a 90% power using a two-sided study with an alpha of 0.05, based on prior published analyses^20,25,26^. We determined a sample size of n = 35 was sufficient for a primary hypothesis testing of RCC stratification of patient’s PFS, with OS stratification as a secondary endpoint. Prior to study initiation, we assumed a drop-out rate of 15% and set a recruitment goal of n = 40 patients. Patients who dropped off the study or were lost to follow up were censored at last known clinical follow up. The blood samples were drawn from both men and women diagnosed with renal cell carcinoma between 2013 and 2015 at the Mayo Clinic Cancer Center. Blood samples were collected from treated and newly diagnosed untreated patients with the primary endpoint being 24 months for progression-free and overall survival. Patients were categorized as having stage 4 (n = 37) or stage 3 (n = 3) disease. Of the 40 patients, 38 patients had clear cell Renal Cell Carcinoma, one had chromophobe RCC, and one had mucinous tubular and spindle cell carcinoma.

Of the 119 blood samples, 79 were taken at varying time points after the baseline sample. 13 patients had 1 follow up sample, 3 patients had 2, 4 patients had 3, 4 patients had 5, 4 patients had 6, 1 patient had 8 and one patient had 10 follow up samples. Six samples failed to go through analysis due to clotting. Full study group characteristics are found in Table 1. Information on the patients and results were kept blinded until the end of the study.

**Table 1:** Patients Demographics at Time of Blood Draw.

Demographic Table for 40 RCC Patients		
Variable	Patients (N = 40)	% Total
Gender		
Male	36	90%
Female	4	10%
Age (median: 66, range: 42–85)		
≥ 60	28	70%
< 60	12	30%
Histology		
ccRCC	38	95%
MTSCC	1	2.5%
Chromophobe RCC	1	2.5%
Stage		
Metastatic	37	92.5%
No metastases	3	7.5%
Number of metastases		
1	4	10%
> 1	22	55%
Unknown	14	35%
Metastatic sites		
Lung	18	45%
Lymph nodes	11	27.5%
Bone	8	20%
Brain	5	12.5%
Liver	5	12.5%
Other (Adrenal, etc.)	14	35%
Therapy		
Pazopanib	15	37.5%
Other*	13	32.5%
Untreated	12	30%
Furhman grade		
1	1	2.5%
2	6	15%
3	13	32.5%
4	9	22.5%
Unknown	11	27.5%
Sarcomatoid histology		
Yes	5	12.5%
No	35	87.5%
Hb (gm/dL of blood)**		
≥ 12	12	30%
< 12	7	17.5%
Platelets (1000/µL of blood)**		
< 150	5	12.5%
> 150	14	35%
Neutrophil (1000/µL of blood)**		
Neutrophil < 7	16	40%
Neutrophil > 7	3	7.5%

*Other treatments in patients include Axitinib (n = 2), Sunitinib (n = 2), Gemcitabine (n = 2), Temsirolimus (n = 2), Sorafenib (n = 2), Bevacizumab (n = 1), Everolimus (n = 1), Pembrolizumab (n = 1).

**Hb, Platelet and Neutrophil counts at time of CAML draw were not available for all patients.

### Blood sample collection

Anonymized blood samples were drawn by standard phlebotomy into CellSave vacutainer tubes and shipped overnight at ambient temperatures for processing at Creatv MicroTech. Samples were run with the CellSieve Microfiltration Assay using a low-pressure vacuum system, as previously described^19–23^. The tubes were checked for 7.5 mL volume of blood and clotting. CellSieve microfilters were used to separate CAMLs from 7.5 mL of blood based on size separation. The blood was first prefixed for 15 min then taken up into a syringe from which it was put through a filter using a vacuum pump for applying constant pressure. After filtration, the microfilter was washed with 3 mL of PBS and put into postfixation for 15 min. Then the microfilter was placed into permeabilization buffer for 15 min. The microfilter was then stained with the CellSieve Enumeration Stain Solution (Creatv MicroTech Inc.), containing cytokeratin 8, 18, and 19, Vimentin, and CD45 antibodies, for 1 h^19–23^. After staining the filters were first washed with 10 mL of PBS/0.1%Tween 20 solution then 3 mL PBS. After washing, the filters were mounted onto glass microscope slides using Flouromount-G with DAPI (Southern Biotech). CAMLs were identified as multinucleated giant myeloid cells with a diameter of ≥ 30um^17,20,21^. CAMLs are defined by their diffused cytokeratin expression and CD45 positivity^17,20,21^. CAMLs contain engulfed epithelial tissue from tumor sites resulting in positive EPCAM expression^17,20,21^.

### Analysis of filters

Imaging and CAML enumeration were performed using and Olympus BX51W1 fluorescent microscope with a Carl Zeiss AxioCam as previously described^15,17,24^. The Zen2011 Blue software was used to process the images and measure cell size using its pre-calibrated size tools. Leica LAS Suite X version 3.7.0.20979 software was also used to process the images and measure cell size using a pre-calibrated size tool for CAML quantification.

### Statistical analysis

Univariate and Multivariate hazard ratios with a statistical significance threshold of p < 0.05 for PFS and OS were calculated with the Cox proportional hazard regression using the MATLAB R2020 software. PFS and OS Kaplan–Meier calculation was done using CAML size and time to progression within the 24-month end point. Progression was defined by the date of baseline blood draw to time of tumor growth by PET/CT scans according to RECIST version 1.1 or evidence of new lesions. Five patients dropped off study before the 24-month endpoint and were censored.

### Ethics approval and consent to participate

The study was run through an agreement with Mayo Clinic Cancer Center, with written informed consent from the patients and with local Institutional Review Board (IRB) approval from Mayo Clinic Cancer Center.

## Results

A total of 119 blood samples were collected from n = 40 RCC patients, including 40 baseline samples prior to the induction of new treatment, n = 40 samples taken ~ 30 days after induction of new line of therapy, and n = 39 follow up samples from additional timepoints. Out of the 40 patients, 38 patients had clear cell renal cell carcinoma (ccRCC), one had mucinous tubular and spindle cell carcinoma (MTSCC), and one had chromophobe renal cell carcinoma (Table 1). Of the 40 patients, 37 had stage 4 metastatic disease and three had non-metastatic stage 3 disease (Table 1). The mean age of the patients was 64 and the range was 42 to 85 (Table 1). After 24 months, 20 patients progressed, 15 patients did not progress, and 5 patients dropped off study. Of the 40 patients, 7 had not been previously treated at blood draw and 33 had received a prior therapy but were progressing on their current regimes (Table 1). Six of the 119 blood samples were unable to be used due to inadequate blood volume (< 7.5 mL) or clotting.

**Figure 1:**
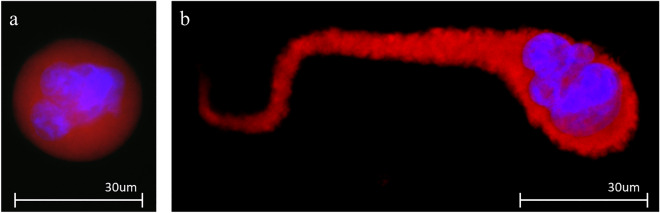
Examples of CAMLs types. (**a**) Example of a small CAML (30 µm in size) from a RCC patient with a multinucleated nucleus as shown by DAPI in blue and cytoplasmic staining with Vimentin (red). (**b**) Example of a large CAML (> 70 µm in size) with an elongated cytoplasmic structure.

**Figure 2:**
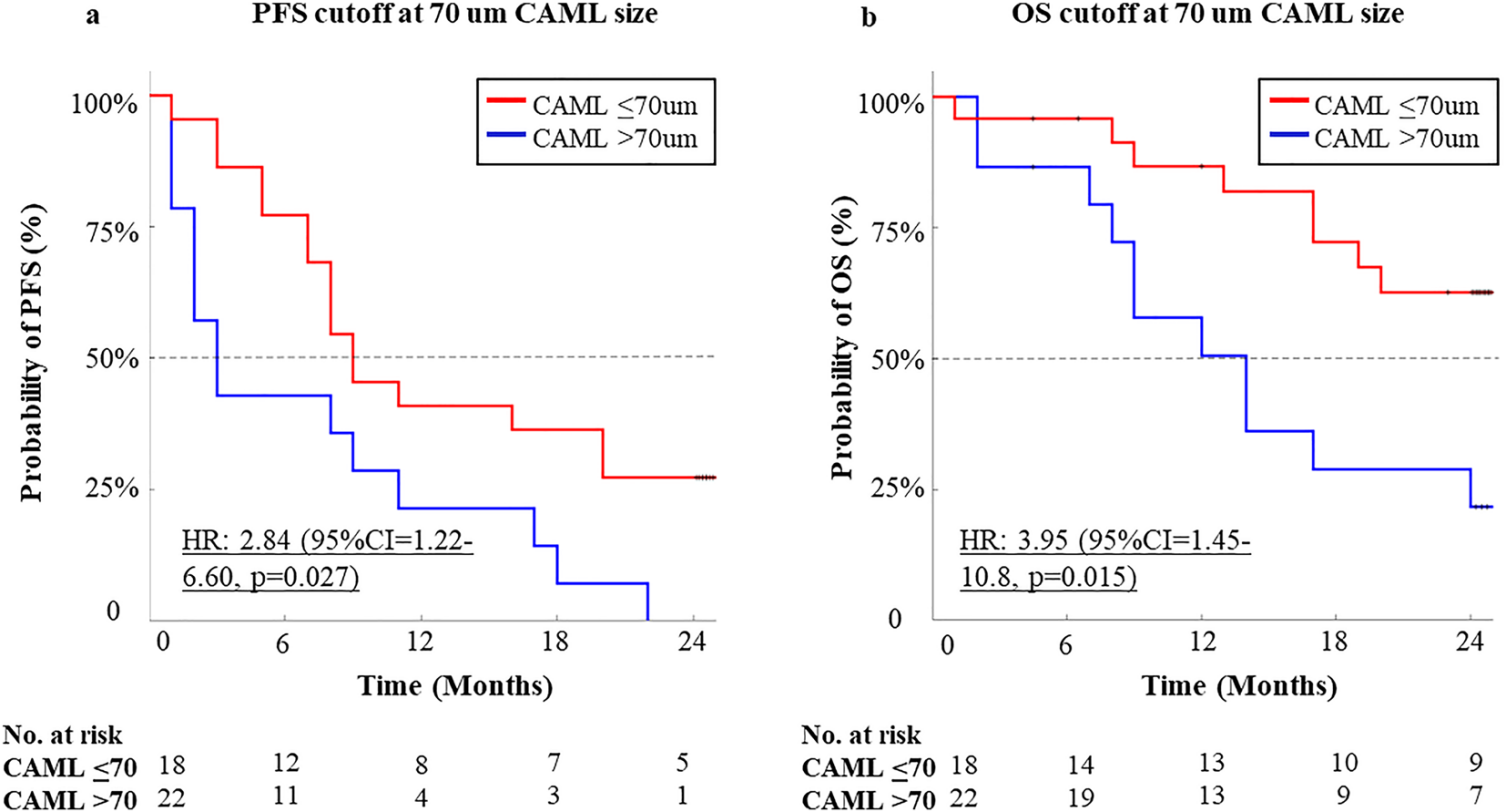
Kaplan-Meiers of PFS and OS. (**a**) PFS of patients with CAMLs ≤ 70 µm vs. > 70 µm. (**b**) OS of patients with CAMLs ≤ 70 µm vs > 70 µm.

All 40 patients had at least one CAML present in their baseline blood sample (Fig. 1) with the average of 5.13 (SD 16.81) per 7.5 mL blood, and the median of 2 (IQR 2). From the total patient population, the average CAML size was found to be 70.08 µm (SD 36.03 µm) and the median was 63 mm (IQR 46.5). Previous studies have shown significance in CAML size (> 50 µm) predicting poorer PFS and OS in patients with metastatic disease^15,20,21^. However, in these RCC analyses, the association between CAML size and hazard ratios of several different CAML sizes ranging from 30 µm to 100 µm (Supplementary Fig. 1), found that a CAML size cutoff of 70 µm was the most optimal option for predicting patient disease progression and overall survival (Fig. 2). Overall, of the patients with CAMLs < 70 µm (n = 10/15) did not progress within 24 months, with n = 2 dropping off study before 24 months, versus patients with CAMLs ≥ 70 µm, (n = 15/22) did progress within 24 months, and 1 dropped off study. The median progression free survival for patients with CAMLs < 70 µm was 8.6 months (95% CI 8.0–19.5) while the median PFS for patients with CAMLs ≥ 70 µm was 2.7 months (95% CI 2.2–10.5). In stratifying clinical outcomes, patients with < 70 µm CAMLs had significantly better overall survival outcomes (HR = 3.95, 95% CI 1.445–10.780, p = 0.0154) as compared to patients with ≥ 70 µm CAMLs (Fig. 2b). Patient PFS outcomes were also significantly better in patients with < 70 µm CAMLs (HR = 2.84, 95% CI 1.220–6.603, p = 0.0273) than with patients with ≥ 70 µm CAMLs (Fig. 2a). One patient was not used in univariate analysis due to dropping off study and therefore lacking survival information.

Change in largest CAML size between baseline blood samples and the blood samples taken at timepoint 2 were compared to determine whether change in CAML size correlated with patient survival outcomes. Change in CAML size from smaller (< 70 µm) to larger (≥ 70 µm) was shown to indicate worse PFS (HR = 5.8, 95% CI 1.56–21.5, p = 0.022) (Supplementary Fig. 2a) but was insignificant in the patients’ OS (HR = 1.3, 95% CI 0.321–5.502, p = 0.975) (Supplementary Fig. 2b). This data suggests that an increase in CAML size was predictive of disease progression and monitoring the change in CAML size may be suggestive of initial treatment response to new lines of therapy.

In a multivariate analysis, run for all known clinical variables in the patient population (Table 2), age was found to be a significant variable, but not an independent variable, while CAML size (p = 0.0224) was found to be the most significant independent predictor for PFS. Additionally, Sarcomatoid, presence of metastases and patients with brain metastasis were all borderline for PFS significance (Table 2). For OS, CAML size (p = 0.0414), Sarcomatoid histology (p = 0.0028), and brain metastases (p = 0.0271) were all significant independent predictors, with Sarcomatoid being the most significant. Patients over the age of 70 had no brain, lung, or bone metastases, however, age was not a significant independent predictor for PFS (p = 0.8699) or OS (p = 0.0985) (Table 2). One patient was not used in the multivariate analysis due to lack of survival data.

**Table 2:** Patients Population Multivariate Analysis.

		PFS				OS		
		Univariate		Multivariate		Univariate		Multivariate
		N*	HR [95%CI]	p-value	p-value	HR [95%CI]	p-value	p-value
CAML size	> 70	24v15	2.91 (1.25–6.79)	**0.02384**	**0.0224**	3.98 (1.46–10.9)	**0.0148**	**0.0414**
CAML number	> 5	34v5	1.35 (0.38–4.74)	0.8880		0.85(0.25–2.89)	0.9556	
Age	> 60	12v27	0.38 (0.20–0.75)	**0.0091**	0.8699	0.23 (0.11–0.50)	**0.0004**	0.0985
Gender	m vs. f	35v4	2.15 (0.77–5.99)	0.2320		1.96 (0.49–7.97)	0.5543	
Sarcamatoid	Yes vs. no	4v35	8.13 (1.38–47.9)	0.0626		17.9 (2.51–128)	**0.0176**	**0.0028**
Neutrophil (1000/µL of blood)**	> 7	3v16	0.59 (0.02–2.23)	0.6625		0.74 (0.11–4.86)	0.8665	
Hb (gm/dL of blood)	> 12	12v7	1.26 (0.41–3.90)	0.9074		0.34 (0.08–1.41)	0.2609	
Platelet (1000/µL of blood)**	> 150	14v5	1.19 (0.36–4.00)	0.9807		2.62 (0.61–11.2)	0.3524	
Stage	4 vs. 3	36v3	0.46 (0.13–1.67)	0.3947		0.32 (0.06–1.78)	0.3868	
Furhman grade**	2 vs. 3	7v13	2.23 (0.88–5.64)	0.1436		2.01 (0.59–6.83)	0.4205	
	2 vs. 4	7v9	2.09 (0.63–6.92)	0.3653		2.49 (0.60–10.2)	0.3682	
	2/3 vs. 4	20v9	1.28 (0.48–3.43)	0.8062		2.04 (0.62–6.76)	0.3862	
	2 vs. 3/4	7v22	2.23 (0.88–5.64)	0.1436		2.01 (0.59–6.81)	0.4205	
Metastatic sites	Yes vs. no	37v2	2.36 (1.05–5.31)	0.0625		2.25 (0.86–5.88)	0.15705	
Lymph nodes metastasis**	Yes vs. no	11v26	0.99 (0.45–2.16)	0.8672		1.05 (0.41–2.68)	0.8826	
Bone metastasis**	yes vs. no	8v29	2.46 (0.87–6.96)	0.1517		3.32 (1.03–10.7)	0.08748	
Brain**	Yes vs. no	5v32	4.71 (1.09–20.3)	0.0882		6.67 (1.44–30.9)	**0.0419**	**0.0271**
Liver**	Yes vs. no	5v32	2.75 (0.75–10.2)	0.2354		1.69 (0.39–7.35)	0.7465	
Lung**	Yes vs. no	18v19	1.45 (0.68–3.08)	0.43617		1.29 (0.52–3.24)	0.7548	

Significant values are in bold.

*n = 1 patient dropped of study and had no follow-up information.

**Information available only for number of patients included in multivariate.

A univariate analysis was run to analyze the significance of hemoglobin, platelets, and WBC (lymphocytes, monocytes, eosinophils, basophils, neutrophils) counts in determining RCC prognosis. Counts were only available for 19 of the 40 patients in this study. Hemoglobin (p = 0.9074), platelets (p = 0.9807), WBC (p = 0.5570), lymphocytes (p = 0.98428), and neutrophils (p = 0.6625) were all insignificant predictors for patients PFS (Supplementary Table 1). Similarly, these blood biomarkers were insignificant in predicting patients OS (Supplementary Table 1). Statistical analysis was not run on monocytes, eosinophils, and basophils due to lack of patients (Supplementary Table 1).

**Figure 3:**
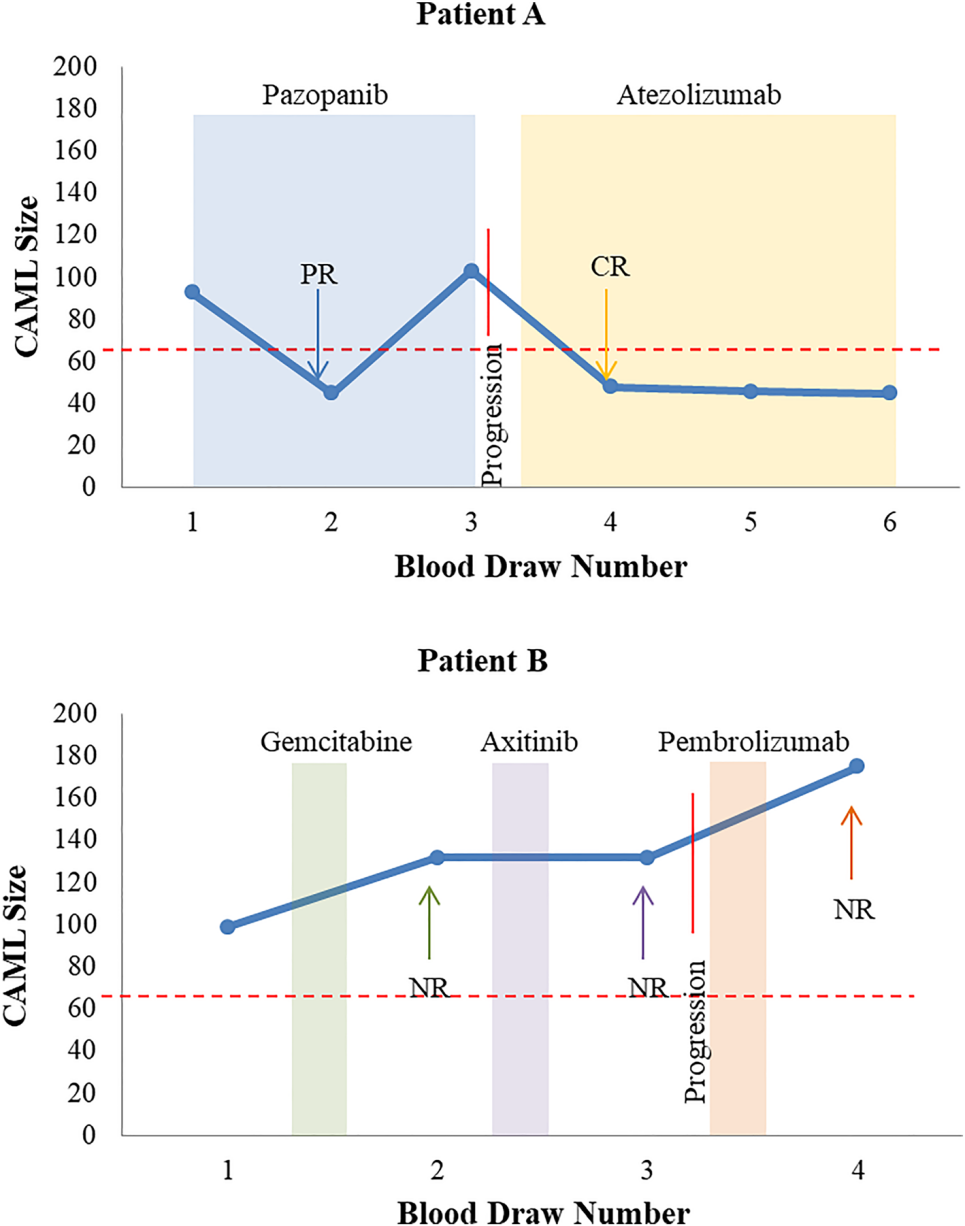
Representative Examples of Tracking the Predictive value of CAML size during patient treatments. (**A**) Patient A had a drop in CAMLs after start of first line therapy, Pazopanib (shaded light blue rectangle), which correlated to a partial response (PR) by PET/CT. This was followed by an increase in CAMLs which correlated with progressive disease. Start of second line therapy, Atezolizumab (shaded yellow rectangle), correlated with a new drop in CAMLs and correlated with complete response (CR). (**B**) Patient B had no response (NR) to first line therapy, Gemcitabine (shaded green rectangle), NR to second line therapy, Axitinib (shaded purple rectangle), and NR to third line therapy, Pembrolizumab (shaded orange rectangle), with CAMLs increasing at every time point. Green, Purple, and Orange arrows = NR. Red line = progression. Red dashed lines = 70 µm CAML threshold. Solid blue circles represent largest CAML sizes at each timepoint. Blue arrow = PR, yellow arrow = CR, red line indicates progression of disease.

Two patients volunteered for multiple follow up blood draws with at least four treatment cycles available for each patient to evaluate the changes of CAMLs over time and treatment inductions. Patient A was treated with Pazopanib during the first three blood draws, then Atezolizumab for the next three blood draws, and had an OS of a minimum of 24 months (Fig. 3A). An initial decrease in CAML size was seen after initial induction of Pazopanib, which appeared to correlate with a partial response. However, after 2 cycles of therapy, CAML size increased at the third blood draw, which then correlated with progressive disease. The patient was then treated with Atezolizumab and a decrease in CAML size was seen in all subsequent blood draws which then correlated with a complete response of the tumor. Patient B was treated with Gemcitabine between the first and second blood draw which correlated with an increase in CAML size and with no response observed by PET/CT (Fig. 3B). A second line therapy of Axitinib was given and further increase of CAML size was observed, also with no response by PET/CT and confirmed progression. A third line therapy of Pembrolizumab was given where the CAML size continued to increase and again no clinical response was observed. The patient then dropped off study and had an OS of 14 months. Overall, these two case studies suggest that monitoring the changes in CAML size is feasible and may coincide with tumor response with new lines of treatment induction. While limited case studies, these results suggest that changes in CAMLs can occur within 1 cycle of therapy (~ 3–4 weeks), which appear to pre-predict the corresponding tumor changes in the PET/CTs, with increases in CAMLs suggestive of no response and decrease in CAMLs suggestive of tumor response to treatment.

## Conclusion

In this study, it was observed that the presence of CAMLs in the blood was a common indicator in patients with advanced RCC malignancy. All RCC patients were found to have at least one CAML in their blood sample at baseline, prior to treatment start for newly diagnosed disease or for patients with progressive disease. Additionally, CAML size was significant in determining PFS (p = 0.027) and OS (p = 0.015) for patients with mRCC. The presence of large CAMLs (> 70 mm) was synonymous to poorer PFS and OS and patients that showed an increase in CAML size after baseline progressed faster than patients that did not. This supports the hypothesis that CAMLs might act as a biomarker for indicating malignant tumors and predicting disease progression, as CAMLs are present at all stages of cancer but not in benign conditions and change in response to new therapy induction.

Many separation techniques have been used to isolate cancer cells, including flow cytometry, CellSearch and RT-PCR. Each of these techniques has its own limitations. Using flow cytometry results in decreased viability of cells and requires separation of cells individually, resulting in a smaller number of cells left to be analyzed^27,32^. CellSearch is heavily dependent on EpCam and therefore does not detect cancerous cells with low EpCam expression^32^. RT-PCR has a high level of false positives due to contamination and expression of genes on normal cells^32^. The CellSieve Microfiltration Assay uses size exclusion to separate cancerous cells with a low level of cell contamination^33^. Even though the CellSearch system is FDA approved, a comparison between CellSearch and the CellSieve Microfiltration Assay shows higher sensitivity and specificity using the latter^33^.

Previous research articles have discussed the role of CAMLs as blood-based biomarkers with a high potential for determining survival outcomes. These studies show that the larger the size and the higher the number of CAMLs found in the patients’ blood the worse PFS and OS^15,20,21^. In this study similar results are obtained, where larger CAMLs means worse PFS and OS as compared to smaller CAMLs. Due to a limited number of patients and a lack of information on treatment response and clinical data, further research needs to be done to validate the results obtained in this study.

Currently, liquid biopsies are gaining popularity in cancer research. The analysis of CTCs, ctDNA/cfDNA, metabolites and exosomes are being used to screen, monitor and diagnose RCC^34^. The use of CTCs has not shown significant potential in determining treatment response or progression in RCC^34^. CtDNA/cfRNA have a low detection rate and are not consistently found in patients with RCC^34,35^. Changes in metabolic profiles can help detect cancer related abnormalities in the biometabolic pathways of individual patients; however, they are not useful in monitoring a general population due to the varying metabolic compounds from one individual to another^34^. Research on exosomes has increased in the past few years, leading to advancements in exosome isolation techniques^34,36,37^. Unfortunately, the various kits available for exosome extraction produce inconsistent results because of the different extraction methods used^34,36,37^. The limitations of the techniques mentioned show that further research in the field of liquid biopsies is needed. CAMLs show great potential as prognostic and diagnostic markers in the field of liquid biopsies as they have been found consistently in patients with various cancers and are efficiently isolated using a size-based filtration technique^15–17,20,21,26^.

In the last few years, numerous new treatments for renal cell carcinoma have been approved; however, there has been a lack of efficiency in the ability to determine the most effective treatment and whether a treatment is no longer effective^9–11^. Hypothetically, blood based biopsies are an effective method to track treatment response (i.e. progression) throughout a patient’s treatment types and may be used as an early indicator of tumor changes^12,15–22^. Currently, the need for a biomarker to help plan the course of treatment for individuals with mRCC is important, as there are numerous adverse side effects of current treatment regimens that may not be beneficial, while numerous alterative treatment options exist^12–14^. Since CAMLs are known to have elements of the primary tumor, a decrease in the size of the tumor, or complete recovery in the tumor should lead to a decrease in the number of CAMLs, as tumor macrophages would have less material to take in through phagocytosis. Here we describe a case study, Patient A, which showed a partial response to a treatment that correlated with a decrease in CAMLs, and subsequently an increase in CAMLs that successfully predicted disease progression. A second case study, Patient B, showed only increases in CAMLs throughout three different treatment types and all increases correlated with lack of tumor response (Fig. 3). With the increase in the number of treatments for advanced RCC, it has become clear there is a need to rapidly identify response, non-response, and drug resistance, as to switch to one of many alterative drug regimes. With the growing incidence of RCC and poor prognosis for individuals with metastatic disease, it is essential to study new predictive and prognostic biomarkers like CAMLs to help patients have better clinical outcomes. Monitoring cells in a blood based biopsy methodology may allow for real time assessment of tumor response and function as a rapid non-invasive method to guide treatment decisions for this purpose. While promising, these initial results clearly require larger and more refined studies, as well as specific interventional trials to determine if CAMLs are truly clinically relevant and if alterations in therapies, based on CAML’s change, effect clinical outcomes in advanced RCC.

## Supplementary Information

Supplementary Figure 1.

Supplementary Figure 2.

Supplementary Table 1.

## Data Availability

The data used in this study and the original cell images supporting Fig. 1, will be made available on reasonable request from Ms. Amama Ali, email address: aali@creatvmicrotech.com.

## Abbreviations

RCC: Renal cell carcinoma
mRCC: Metastatic renal cell carcinoma
ccRCC: Clear cell renal cell carcinoma
MTSCC: Mucinous tubular and spindle cell carcinoma
CAML: Cancer associated macrophage-like cell
PFS: Progressive free survival
OS: Overall survival
HR: Hazard ratio
CI: Confidence interval
IMDC: International mRCC database consortium
IRB: Institutional review board
WBC: White blood cell

## Code availability

Statistical analysis was done using MATLAB R2020b (The MathWorks Inc, Natick, Massachusetts, USA). Codes for statistical analysis were KMplot (https://github.com/dnafinder/kmplot) and Logrank (https://github.com/dnafinder/logrank).
