# Supplementary figures and images for: Cancer associated macrophage-like cells in metastatic renal cell carcinoma predicts for poor prognosis and tracks treatment response in real time
Source: Sci Rep. 2023 Jun 29;13:10544. doi: 10.1038/s41598-023-37671-3 (PMC10310728; doi:10.1038/s41598-023-37671-3)

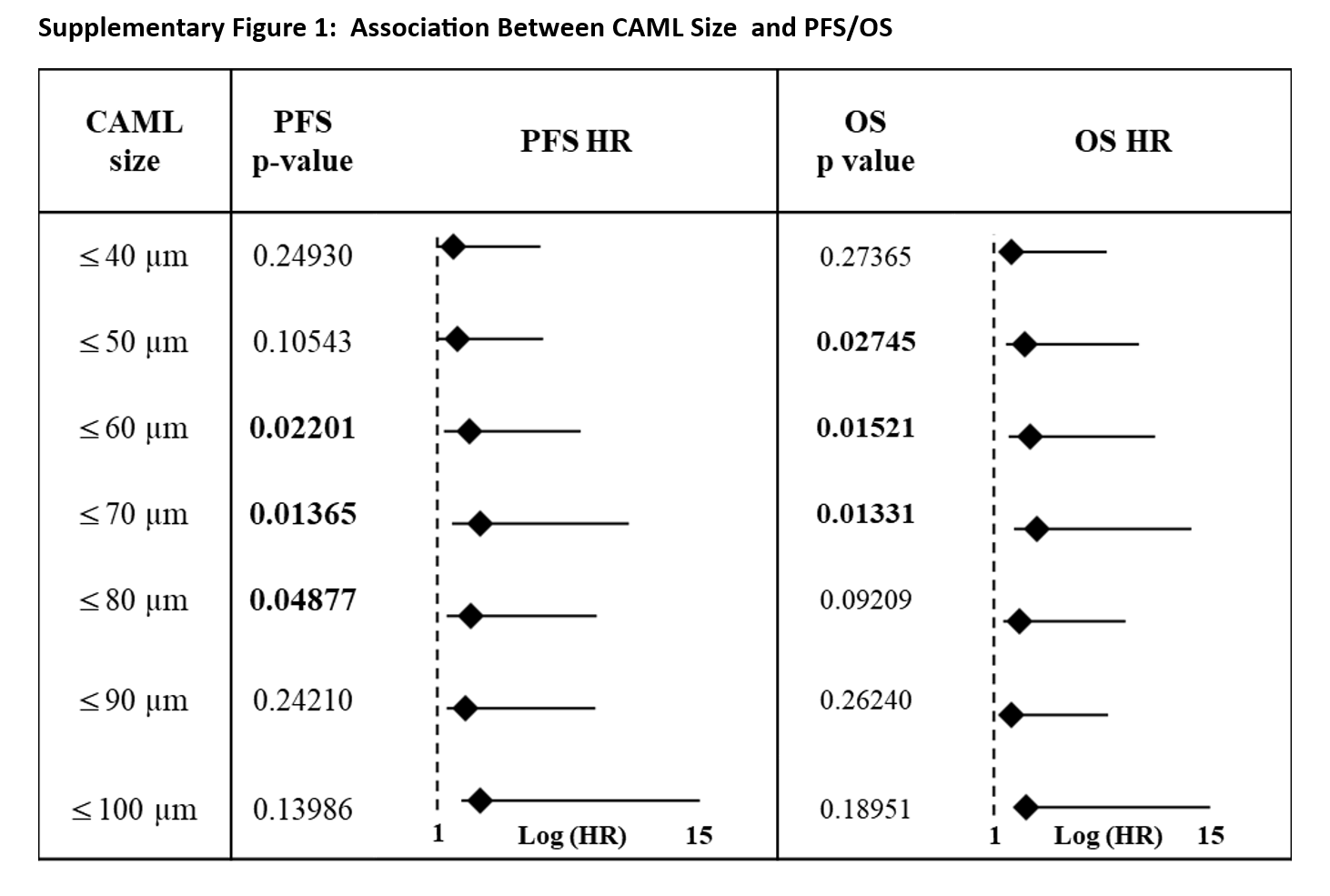

Supplement: Supplementary file 1 — Supplementary Figure 1. [file 41598_2023_37671_MOESM1_ESM.tif]

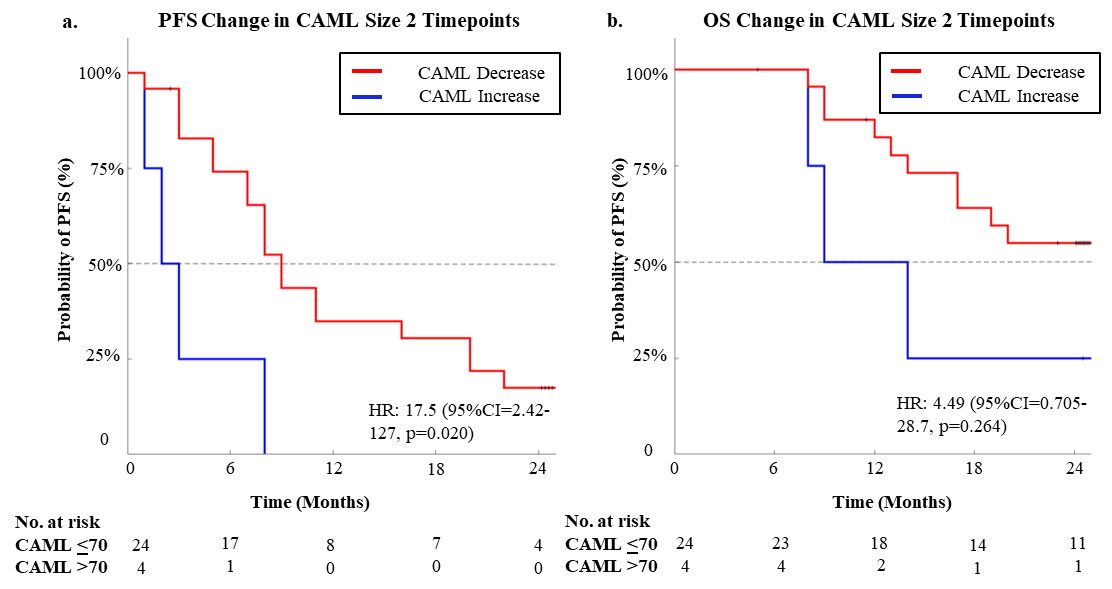

Supplement: Supplementary file 2 — Supplementary Figure 2. [file 41598_2023_37671_MOESM2_ESM.tif]
