# Supplementary material for: Cancer associated macrophage-like cells in metastatic renal cell carcinoma predicts for poor prognosis and tracks treatment response in real time
Source: Sci Rep. 2023 Jun 29;13:10544. doi: 10.1038/s41598-023-37671-3 (PMC10310728; doi:10.1038/s41598-023-37671-3)
Supplement: Supplementary file 3 — Supplementary Table 1. [file 41598_2023_37671_MOESM3_ESM.docx]

**Supplementary Table 1 Patients Population Blood Biomarkers and Relative PFS/OS**

|  | **PFS** | **OS** |
| --- | --- | --- |
|  | **Univariate** | **Univariate** |
|  | **N HR[95%CI] p-value** | **HR[95%CI] p-value** |
| **WBC (4500-11000/uL of blood)*** | 4v15 0.5(0.1-2.1) 0.5570 | 1.3(0.3-6.0) 0.99174 |
| **Lymphocytes (1000-4800/uL of blood)*** | 6v13 1.2(0.4-3.7) 0.98428 | 1.2(0.3-5.1) 0.88279 |
| **Monocytes (800/uL of blood)*** | 1v18 N/A N/A | N/A N/A |
| **Eosinophils (500/uL of blood)*** | 1v18 N/A N/A | N/A N/A |
| **Basophils (300/uL of blood)*** | 0v18 N/A N/A | N/A N/A |
| **Neutrophil (1000/uL of blood)* >7** | 3v16 0.59(0.02-2.23) 0.6625 | 0.74(0.11-4.86) 0.8665 |
| **Hb (gm/dL of blood)* >12** | 12v7 1.26(0.41-3.90) 0.9074 | 0.34(0.08-1.41) 0.2609 |
| **Platelet (1000/uL of blood)* >150** | 14v5 1.19(0.36-4.00) 0.9807 | 2.62(0.61-11.2) 0.3524 |

*Information available only for number of patients included in univariate
